# SRM: A Source-Reprojection Module for Cross-Day sEMG Gesture Recognition

**DOI:** 10.3390/s26123870

**Published:** 2026-06-18

**Authors:** Dian Li, Peiji Chen, Shunta Togo, Hiroshi Yokoi, Yinlai Jiang

**Affiliations:** 1 Department of Mechanical and Intelligent Systems Engineering, The University of Electro-Communications, Tokyo 182-8585, Japan; li.dian@uec.ac.jp (D.L.); chen.peiji@uec.ac.jp (P.C.); s.togo@hi.mce.uec.ac.jp (S.T.); yokoi@hi.mce.uec.ac.jp (H.Y.); 2 Center for Neuroscience and Biomedical Engineering, The University of Electro-Communications, Tokyo 182-8585, Japan

**Keywords:** cross-day adaptation, domain shift, gesture recognition, source-free adaptation, surface electromyography, unsupervised domain adaptation

## Abstract

Surface electromyography (sEMG) gesture recognition degrades across recording days under domain shift, increasing calibration burden for myoelectric interfaces. Many cross-day adaptation pipelines retrain the deployed recognizer or require labeled target-session data, which can be impractical in assistive-device settings where classifier versions may need to remain locked for traceability and regulatory compliance. We study unsupervised cross-day adaptation under two constraints: the task classifier remains frozen and holdout-day labels are not used when training the adaptor. We propose the Source-Reprojection Module (SRM), a plug-in front end that combines conditional adversarial feature learning with a residual signal-space projector guided by the frozen classifier’s gradients, identity regularization, and latent-space distribution matching, using labeled source days and unlabeled adaptation days only. On a multi-day protocol with four healthy participants (at least five calendar-day sessions per participant, split 3:1:1 into source, adaptation, and holdout domains) and three random seeds per participant (12 runs), mean holdout accuracy increases from 70.9% for the frozen classifier alone to 72.8% with SRM (+1.98±0.91 percentage points averaged across subjects). SRM outperforms the frozen baseline in 10 of 12 subject–seed runs. The gain is modest and the cohort is small, so the result supports proof-of-mechanism under the stated protocol rather than population-level clinical generalization.

## 1. Introduction

Surface electromyography (sEMG) captures the electrical activity of skeletal muscles and serves as a primary input modality for myoelectric prostheses [1,2,3,4], exoskeleton control [5,6], and rehabilitation interfaces [7,8]. Complementary sensing modalities are also being integrated into prostheses to relax exclusive reliance on muscular signals under difficult conditions [9]. This paper focuses on wearable multi-channel sEMG to keep the acquisition stack compact and regulation boundaries explicit. A widely used approach to sEMG-based gesture recognition relies on pattern recognition classifiers trained on labeled data collected during an initial calibration session [10,11]. However, classifier performance degrades notably when applied to recordings from subsequent days or sessions due to domain shift, i.e., systematic distributional changes between training and deployment conditions [12,13,14,15]. In practice, this instability forces users to undergo repeated calibration procedures, increasing the burden on patients and clinicians and contributing to high prosthesis abandonment rates [16].

The sources of cross-day domain shift are multifactorial: electrode displacement alters channel-to-muscle mappings; skin impedance varies with hydration and temperature; and neuromuscular fatigue or adaptation changes the underlying signal statistics [17,18,19]. Among these, the core difficulty is that such shifts are non-stationary and subject-specific, making it infeasible to pre-characterize or adequately compensate them at design time [20,21,22].

**Figure 1:**
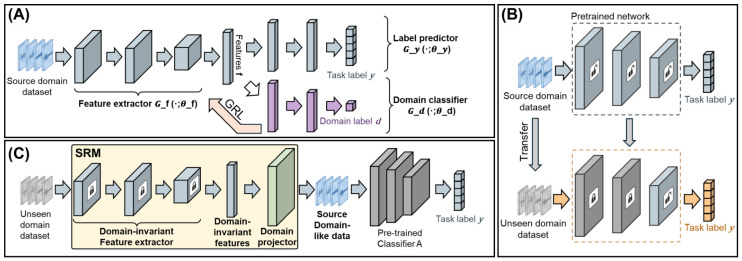
Structural comparison of three adaptation paradigms for cross-day sEMG gesture recognition: (**A**) end-to-end domain adaptation with joint updates to the feature extractor and classifier (e.g., adversarial or maximum mean discrepancy (MMD)-based alignment); adversarial variants usually insert a gradient reversal layer (GRL) between the feature extractor and the domain critic to implement the min–max objective [32], (**B**) Transfer Learning/fine-tuning, and (**C**) the proposed Source-Reprojection Module (SRM) inserted before frozen classifier *A*. The key distinction is that SRM preserves classifier *A* while using unlabeled adaptation data.

To address this problem, existing cross-session sEMG adaptation methods can be broadly divided into two categories. (i) End-to-end domain adaptation methods [12,23] typically employ adversarial or distribution-matching objectives to retrain the model and learn domain-invariant representations. (ii) Transfer Learning and fine-tuning strategies [24,25,26] adapt selected layers of an existing model using a small amount of labeled target data, or alternatively employ pseudo-labeling for source-free adaptation [27,28,29]. Figure 1 illustrates the structural distinction between these paradigms and the proposed approach.

However, these mainstream paradigms may be difficult to reconcile with stringent medical-device deployment requirements. Under regulatory frameworks such as the U.S. Food and Drug Administration (FDA) and the European Union Medical Device Regulation (MDR), AI-based Software as a Medical Device (SaMD) is often expected to be deployed in a locked state, where arbitrary post-deployment updates to core model parameters may trigger additional review or re-validation procedures [30]. From this perspective, both paradigms above rely on modifying the deployed recognition model during adaptation. In addition, label-dependent fine-tuning introduces a recurring calibration burden that may disrupt practical use [27], while updating unfrozen network parameters can also increase the risk of catastrophic forgetting or unstable negative transfer [31].

Therefore, the goal of this study is to improve recognition from wearable multi-channel sEMG acquired on different days and sessions, when channel–muscle coupling and skin–electrode conditions change between recordings and induce input non-stationarity. We seek to do so under two deployment-aligned constraints: the deployed classifier weights must remain unchanged, and repeated per-session recalibration on labeled holdout data should be avoided. Specifically, we study the following problem: given a pre-trained classifier *A* that is fitted to source-day data but degrades on cross-day target data, can we recover its holdout-day performance without modifying *A* and without requiring any holdout-day labels?

To this end, we propose a classifier-preserving pre-alignment framework that places a trainable Source-Reprojection Module (SRM) before classifier *A*. The module consists of two components trained in sequence: (i) a domain-robust feature extractor learned via conditional adversarial alignment using labeled source data and unlabeled target data; and (ii) a residual signal-space projector *P* that transforms target inputs toward the source distribution, guided by the frozen classifier’s gradients and regularized by identity preservation and distribution matching. Because classifier *A* remains frozen throughout, the approach satisfies the classifier-preservation constraint; because only unlabeled adaptation data are required, it also removes the recurring labeling burden. SRM is implemented as a front-end module and can be inserted or removed without retraining classifier *A*. Section 3 reports experiments on a multi-day sEMG protocol under frozen classifier *A*, together with comparisons to controlled baselines and to adaptation strategies that update the deployed classifier.

The main contributions are twofold. (i) We propose a plug-in pre-alignment framework that combines conditional adversarial feature learning with a residual signal-space projector guided by the frozen classifier’s gradients, thereby satisfying both the label-free and classifier-preservation constraints. (ii) We evaluate this framework on a subject-wise multi-day wearable sEMG protocol with explicit day/session splits, contrasting holdout performance under frozen *A* against baselines and against end-to-end alternatives that relax the frozen-classifier assumption.

The remainder of this paper is organized as follows: Section 2 presents the proposed method; Section 3 reports experimental results; Section 4 discusses findings, generality, and limitations; and Section 5 concludes the paper.

## 2. Materials and Methods

### 2.1. Data Acquisition, Preprocessing, and Windowing

We collected sEMG data from four healthy adult males (aged 24–28). Handedness was recorded (three right-handed, one left-handed). All recordings were acquired from the right forearm over the extensor muscle group using surface electrodes. This study was approved by the Research Ethics Committee of The University of Electro-Communications (Approval No. H10006(7)), and all participants provided written informed consent prior to the experiments.

**Figure 2:**
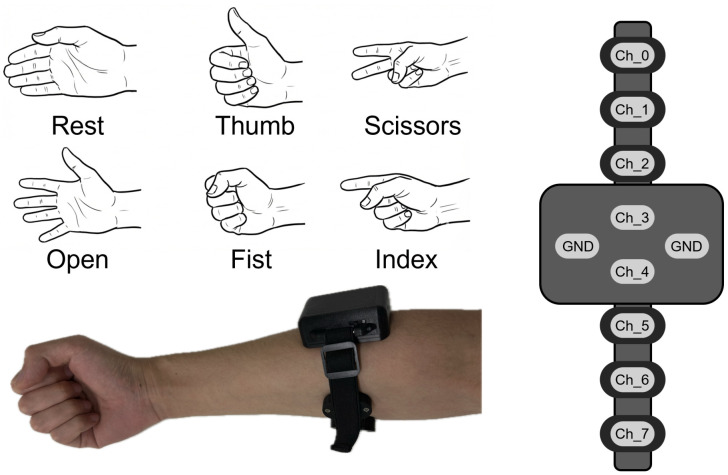
MindRove eight-channel forearm band and six-gesture set used for multi-session acquisition; overlapping extensor activation patterns motivate cross-day confusion under electrode placement variability (cf. day-split evaluation in Section 3).

Signals were recorded using the MindRove armband (model ARB.210901; MindRove, Budapest, Hungary), a wearable Wi-Fi-connected device equipped with eight conductive-fabric semi-dry sEMG electrodes and two auxiliary electrodes (reference and bias), providing C=8 active EMG channels sampled at 500 Hz. The armband was placed on the dominant forearm, centered over the proximal extensor muscle group, with the electrode array encircling the limb circumferentially [33,34]. Figure 2 illustrates the recording setup and the six gesture classes used in this study. The circumferential arrangement captures both dorsal and volar muscle groups, providing complementary activation patterns for multi-finger gesture discrimination.

The gesture set was designed with reference to the NinaPro benchmark database [35,36], selecting a subset of six postures that spans a representative range of functional hand configurations: gross power grips (Fist, Open hand), precision postures (Scissors, Index pointing, Thumb-up), and a rest state. Several pairs share overlapping forearm extensor activation patterns (e.g., Scissors vs. Index, Open vs. Fist), making them susceptible to inter-day confusions caused by electrode displacement [37]. This fixed vocabulary intentionally omits dedicated wrist-DOF gestures and fine-pinch taxonomies (e.g., pinch and lateral grasps) so that this study isolates forearm-band discrimination under cross-day shift; extending the label set and sensing layout for multi-DoF prosthetic wrists is left to future work.

Each session comprised the six gesture classes described above. Gestures were prompted via on-screen images displayed in fixed order. For each prompt, the participant assumed and maintained the target posture; recording began after posture stabilization and lasted 4 s, followed by a rest period before the next prompt.

For each subject, we recorded ≥5 distinct domains corresponding to different recording dates/sessions, reflecting realistic attachment variations (e.g., slight placement differences and tightness). Domains are indexed by subject ID and acquisition date.

Raw sEMG was processed per channel with detrending (constant), a 2nd-order Butterworth band-pass filter (center 125 Hz, bandwidth 250 Hz), and a 2nd-order Butterworth band-stop filter (center 5 Hz, bandwidth 10 Hz).

Gesture prompting and sEMG recording used a custom Python 3.12.11 interface with the MindRove Python SDK (mindrove 3.0.1; BrainFlow 5.22.1). The Butterworth filters above were applied with MindRove DataFilter during trial preprocessing. Holdout accuracy, SRM training, and ablation runs were using PyTorch 2.7.1, NumPy 2.4.6, scikit-learn 1.7.1, and Matplotlib 3.10.5.

Each 4 s trial at 500 Hz yields 2000 samples. We use a sliding window of T=100 samples (0.2 s) with a step size of 25 samples (0.05 s), producing 77 windows per trial. Windows are stored as individual .npz files with an array of shape (8,100) and a consistent label mapping across all subjects and domains.

### 2.2. Problem Formulation and Day-Split Protocol

We study within-subject sEMG gesture recognition under cross-day/cross-session domain shift. Let each sEMG sample be a multi-channel segment $x\in R^{C\times T}$ with *C* channels and *T* time steps.

We retain the assumption stated in the Introduction: classifier A(·) is trained on labeled source days but degrades on unseen days due to domain shift. Our goal is to improve performance on unseen test days without retraining classifier *A* and without using labels from those days.

For each subject, we partition recording sessions across multiple days into three disjoint sets: (i) source days $D_{\mathrm{src}}=\left\{\left(x_{i},y_{i}\right)\right\}$, which provide the labeled data used to train classifier *A* and to supply supervised signals for SRM; (ii) adaptation days $D_{\mathrm{adapt}}=\left\{x_{j}\right\}$, which provide unlabeled intermediate-day data for SRM training; and (iii) holdout test days $D_{\mathrm{test}}=\left\{\left(x_{k},y_{k}\right)\right\}$, which are reserved exclusively for evaluation.

In a typical configuration, we use a 3:1:1 split across source/adaptation/holdout test days. The adaptation days provide unlabeled samples that reflect realistic day-to-day variability, enabling unsupervised alignment without requiring labeled calibration data from the holdout test days.

### 2.3. Framework Overview

**Figure 3:**
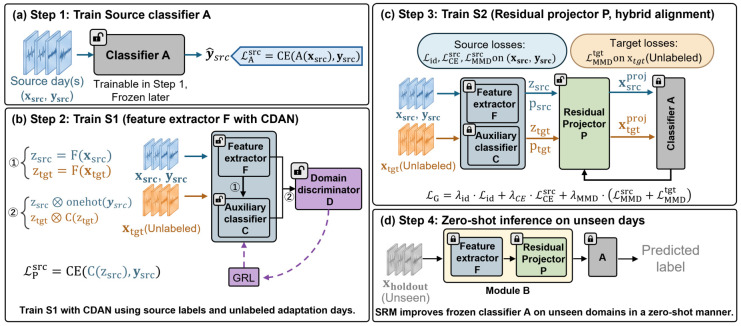
Overview of the proposed classifier-preserving training schedule (read left-to-right): Step 1 fits and freezes classifier *A* only on source-day labels. Step 2 fits SRM component $S_{1}$ (*F*, *C*, and *D*) with labeled sources and unlabeled adaptation days. Step 3 fits projector *P* while freezing *A*, *F*, and *C*. Holdout evaluation (Step 4) applies the cascade $S_{1}\to P\to A$ (not “*A* before SRM”) on unseen test days.

Figure 3 illustrates the overall pipeline of the proposed cross-day sEMG gesture recognition framework. The full procedure consists of three training stages and one testing stage. First, classifier *A* is trained on source days and then frozen. Second, we decompose the Source-Reprojection Module (SRM) as $S=(S_{1},S_{2})$, where $S_{1}$ is trained on labeled source data and unlabeled adaptation data to extract class-relevant latent features that are less sensitive to day-specific variation, without updating classifier *A*. Third, $S_{2}$ is trained to reproject the input signal toward a representation that is more compatible with frozen classifier *A* while keeping classifier *A* and $S_{1}$ fixed. During the testing stage, the learned SRM is placed before classifier *A*, and a holdout sample is classified without any additional adaptation. The following subsections provide the stage-specific objectives and training details.

### 2.4. Stage A: Training Classifier *A*

We begin by training classifier *A* on labeled source days $D_{\mathrm{src}}$ using standard supervised learning with cross-entropy loss:

(1) $$\min_{A}\;E_{\left(x,y\right)∼D_{\mathrm{src}}}\;\mathrm{CE}\left(A(x),y\right).$$

In our implementation, classifier *A* is a dilated temporal convolutional network with eight input channels following the TCN-style dilated residual design [38], trained with Adam [39] (learning rate $1\times 10^{-3}$, batch size 32). We terminate Stage A training after 10–12 epochs per subject—the schedule was chosen empirically after observing diminishing validation accuracy improvements while retaining stable train versus validation gaps in our monitoring logs.

For each subject, we split $D_{\mathrm{src}}$ into train/validation subsets (80/20 stratification with fixed seeds) throughout Stage A optimization. Classification accuracy and cross-entropy on the validation split are recomputed whenever the optimizer finishes an epoch over the entire training shard; checkpoints are persisted on disk for later B1/B2 stages. We deliberately avoid early stopping in the reported baseline so that successive pipeline stages consume identical artefacts across random seeds without introducing an auxiliary hyperparameter search solely for Stage A—validation curves nevertheless confirm that divergence between training and validation accuracy stays moderate for typical subjects. Upon completion, classifier *A* is frozen and never updated in subsequent stages.

### 2.5. Stage S1: Learning Domain-Robust Latent Features

In Stage S1, SRM component $S_{1}$ learns the latent representation used in the subsequent reprojection stage. It consists of a feature extractor *F*, an auxiliary classifier *C*, and a domain discriminator *D* in a Conditional Domain Adversarial Network (CDAN) configuration [40,41,42]. For an input signal *x*, Stage S1 produces the latent feature *z* and the auxiliary prediction $\hat{p}$ as

(2) $$z=F\left(x\right),\;\hat{p}=C\left(z\right).$$

Here, *z* is the shared latent representation used by the auxiliary classifier and the domain discriminator, and it is also passed to Stage S2 as a conditioning signal. To discourage the domain discriminator from matching marginals while ignoring semantic structure, CDAN [40] pairs every latent dimension with auxiliary class probabilities: we instantiate $\phi \left(z,\hat{p}\right)=z⊗\mathrm{softmax}\left(\hat{p}\right)$, yielding a flattened vector that intertwines semantic and domain cues—only samples that preserve class-conditioned statistics become indistinguishable across domains under *D*.

To preserve task discriminability, we train *C* to classify source samples correctly:

(3) $$L_{\mathrm{cls}}=E_{\left(x,y\right)∼D_{\mathrm{src}}}\;\mathrm{CE}\left(C(F(x)),y\right).$$

Let $\tilde{z}=\phi \left(z,\mathrm{softmax}(\hat{p})\right)$ where z=F(x) and $\hat{p}=C\left(z\right)$ denote logits feeding both the outer product and the softmax in ϕ. The domain discriminator *D* is trained to distinguish source from adaptation samples:

(4) $$\begin{array}{cc} L_{\mathrm{dom}} & =E_{x∼D_{\mathrm{src}}}\;\mathrm{CE}\left(D(\tilde{z}),0\right)\\ \; & \;+E_{x∼D_{\mathrm{adapt}}}\;\mathrm{CE}\left(D(\tilde{z}),1\right), \end{array}$$

where domain labels 0/1 indicate source/adaptation days.

The Stage S1 optimization forms an adversarial game between the feature-learning branch (F,C) and the domain discriminator *D*: *D* is trained to distinguish the two domains, while *F* and *C* are trained to preserve source-domain class discrimination and reduce domain-specific information in the latent representation. Concretely, we optimize

(5) $$\min_{D}L_{\mathrm{dom}},$$

and

(6) $$\min_{F,C}\max_{D}\;\left(L_{\mathrm{cls}}-\lambda_{\mathrm{dom}}L_{\mathrm{dom}}\right).$$

In practice, the adversarial interaction between *F* and *D* is implemented by inserting a gradient reversal layer (GRL), which scales gradients by a negative coefficient when back-propagating from *D* to *F*, equivalent to reversing the ascent direction inside the saddle-point objective [32]. Consequently, *D* is optimized to discriminate domains while (F,C) receive opposing gradients that incentivize latent features preserving class discriminability yet suppressing separable domain structure.

We train *F*, *C*, and *D* jointly on source days $D_{\mathrm{src}}$ (labeled) and adaptation days $D_{\mathrm{adapt}}$ (unlabeled). In our implementation, we use AdamW optimizer with learning rate $1\times 10^{-3}$, weight decay $1\times 10^{-4}$, batch size 64, and train for 10–15 epochs. We set $\lambda_{\mathrm{dom}}=2.0$ and use a warmup schedule for the domain adversarial loss (first five epochs focus on classification, then gradually increase adversarial weight).

After Stage S1, we save the trained *F* and *C* and freeze them for the next stage.

### 2.6. Stage S2: Residual Signal-Space Alignment Guided by Frozen Classifier *A*

In Stage S2, SRM component $S_{2}$ is implemented as a signal-space residual projector *P*. Given an input signal *x*, frozen Stage S1 first produces the latent feature and auxiliary prediction in Equation (2). Stage S2 then generates an aligned signal by

(7) $$x_{\mathrm{aligned}}=x+\Delta x,\;\Delta x=P\left(x,z,\hat{p}\right),\;z=F\left(x\right),\;\hat{p}=C\left(z\right).$$

Unlike a feature-space projector, *P* outputs a residual correction in the original signal space—here $\left(z,\hat{p}\right)$ are the Stage S1 outputs defined in Equation (2). The aligned signal is then either classified by classifier *A* or re-encoded by *F*, depending on the loss term.

We require that aligned signals remain classification-friendly for the frozen classifier *A* on labeled source samples:

(8) $$L_{A-\mathrm{CE}}=E_{\left(x,y\right)∼D_{\mathrm{src}}}\;\mathrm{CE}\left(A(x_{\mathrm{aligned}}),y\right),$$

where z=F(x), $\hat{p}=C\left(z\right)$, and $x_{\mathrm{aligned}}=x+P\left(x,z,\hat{p}\right)$ is identical to Equation (7). Gradients from *A* anchor *P* to the classifier’s decision boundaries on source data; unlabeled adaptation samples influence *P* only through the MMD [43,44,45] and identity regularizers described below.

To avoid unnecessary distortion of already well-performing source signals, we regularize the alignment to be close to identity on source data:

(9) $$L_{\mathrm{id}}=E_{x∼D_{\mathrm{src}}}\;{∥x_{\mathrm{aligned}}-x∥}_{2}^{2}:=E_{x∼D_{\mathrm{src}}}\;{∥\Delta x∥}_{2}^{2}.$$

We use MSE because the squared penalty provides a stronger gradient signal when source-domain projections deviate substantially from zero, a property that is important during the initial training phase to anchor the projector output close to the identity mapping. To prevent this constraint from dominating later epochs and forcing *P* toward a trivial identity function, we apply a warmup decay schedule: $\lambda_{\mathrm{id}}$ starts at its full value and decays linearly to zero over the first few epochs (three warmup epochs in our setting). This two-phase design first stabilizes the projector by penalizing large deviations from the input, then releases the identity constraint so that *P* can learn non-trivial domain-adaptive corrections.

For unlabeled $D_{\mathrm{adapt}}$, we reduce the domain gap by matching distributions in the latent space of the frozen *F*. The key insight is that the projector *P* operates in signal space, producing aligned signals $x_{\mathrm{aligned}}$ that are then re-encoded by the frozen feature extractor *F* to obtain latent representations. This creates a two-stage transformation: $x\to x_{\mathrm{aligned}}\to z^{\prime }=F\left(x_{\mathrm{aligned}}\right)$, where we constrain the output features $z^{\prime }$ rather than directly manipulating them.

**Figure 4:**
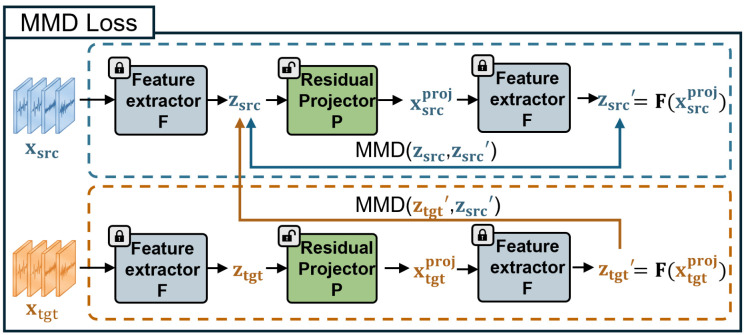
Dual-path maximum mean discrepancy (MMD) alignment in Stage S2. Residual projector *P* emits aligned windows that are mapped by the same frozen extractor *F* as the untouched windows (weights shared across both passes). One empirical MMD term pairs $F(x^{s})$ with $F(x_{\mathrm{aligned}}^{s})$ on source batches; another pairs adaptation projections with corrected sources to close domain gaps.

Figure 4 illustrates the dual-path MMD computation used in Stage S2. For both source and adaptation domains, input signals pass through the frozen feature extractor *F*, then through the trainable projector *P* (which outputs projected signals in the original signal space), and finally through *F* again to obtain the projected latent representations. This architecture enables two complementary MMD constraints:

Let $x_{\mathrm{aligned},i}^{s}$ and $x_{\mathrm{aligned},j}^{a}$ denote aligned signals from source and adaptation days. After re-encoding them with the frozen feature extractor *F*, we obtain

(10) $${\bar{z}}_{i}^{s}=F\left(x_{\mathrm{aligned},i}^{s}\right),\;{\bar{z}}_{j}^{a}=F\left(x_{\mathrm{aligned},j}^{a}\right).$$

We use $\bar{z}$ to distinguish these re-encoded projected features from the original latent feature z=F(x) defined in Equation (2).

For source identity preservation, we compute an MMD between the original source features $z_{\mathrm{orig}}^{s}=F\left(x^{s}\right)$ and the re-encoded projected source features ${\bar{z}}^{s}=F\left(x_{\mathrm{aligned}}^{s}\right)$ to prevent the projector from distorting source-domain signals that are already well-suited for the frozen classifier:

(11) $$L_{\mathrm{MMD}}^{\mathrm{src}}=\mathrm{MMD}\left(F\left(x^{s}\right),F\left(x_{\mathrm{aligned}}^{s}\right)\right).$$

This term, combined with the MSE identity loss (Equation (9)), ensures that the projector acts as a small residual correction on source data rather than a drastic transformation.

For cross-domain distribution alignment, we minimize the MMD between projected adaptation features and projected source features:

(12) $$L_{\mathrm{MMD}}^{\mathrm{tgt}}=\mathrm{MMD}\left(F\left(x_{\mathrm{aligned}}^{a}\right),F\left(x_{\mathrm{aligned}}^{s}\right)\right).$$

This unsupervised loss encourages *P* to transform adaptation-day signals such that, after re-encoding by *F*, they occupy the same region of feature space as the (minimally perturbed) source signals.

The combined MMD objective is:

(13) $$L_{\mathrm{MMD}}=L_{\mathrm{MMD}}^{\mathrm{src}}+L_{\mathrm{MMD}}^{\mathrm{tgt}},$$

where each two-sample term is computed using the standard empirical MMD

(14) $$\begin{array}{cc} \mathrm{MMD}(U,V)= & \frac{1}{n^{2}}\sum_{i,i^{\prime }}k\left(u_{i},u_{i^{\prime }}\right)\\ \; & +\frac{1}{m^{2}}\sum_{j,j^{\prime }}k\left(v_{j},v_{j^{\prime }}\right)-\frac{2}{nm}\sum_{i,j}k\left(u_{i},v_{j}\right), \end{array}$$

with $U={\left\{u_{i}\right\}}_{i=1}^{n}$, $V={\left\{v_{j}\right\}}_{j=1}^{m}$, and k(·,·) denoting a radial basis function (RBF) kernel. The dual-path design (Figure 4) ensures that *P* learns to selectively correct domain-shifted signals while preserving the structure of well-aligned source signals.

We train *P* by combining the three loss terms:

(15) $$\begin{array}{cc} \min_{P}\; & \lambda_{\mathrm{ce}}\;L_{A-\mathrm{CE}}+\lambda_{\mathrm{id}}\;L_{\mathrm{id}}+\lambda_{\mathrm{mmd}}\;L_{\mathrm{MMD}}, \end{array}$$

where $\lambda_{\mathrm{ce}}$, $\lambda_{\mathrm{id}}$, and $\lambda_{\mathrm{mmd}}$ are loss weights.

We train *P* on source days $D_{\mathrm{src}}$ (labeled) and adaptation days $D_{\mathrm{adapt}}$ (unlabeled) with frozen classifier *A*, *F*, and *C*. In our implementation, we use AdamW optimizer with learning rate $1\times 10^{-3}$ for *P*, batch size 64, and train for 10 epochs. For the reported Stage S2 setting, we use $\lambda_{\mathrm{ce}}=2.0$, $\lambda_{\mathrm{id}}=0.1$ (with three-epoch linear warmup decay to zero), and $\lambda_{\mathrm{mmd}}=0.5$. Gradient clipping (max norm 5.0) is applied for stability.

### 2.7. Training and Testing Protocol

For each subject, the complete training pipeline consists of three stages executed sequentially: (1) Train classifier *A* on source days $D_{\mathrm{src}}$ using supervised learning. Freeze classifier *A* after training. (2) Train stage S1. Train feature extractor *F*, auxiliary classifier *C*, and domain discriminator *D* on $D_{\mathrm{src}}$ (labeled) and adaptation days $D_{\mathrm{adapt}}$ (unlabeled) using the CDAN objective (Equation (6)). Freeze *F* and *C* after training. (3) Train stage S2. Train residual projector *P* with frozen classifier *A*, *F*, and *C* on $D_{\mathrm{src}}$ (labeled) and $D_{\mathrm{adapt}}$ (unlabeled) using the combined objective (Equation (15)). Save the trained *P*.

At testing stage, a holdout sample is passed through the trained SRM and then classified by frozen classifier *A*. For an input window *x* from $D_{\mathrm{test}}$, we: (1) compute the Stage S1 outputs z=F(x) and $\hat{p}=C\left(z\right)$; (2) apply Stage S2 to obtain the aligned signal: $x_{\mathrm{aligned}}=x+P\left(x,z,\hat{p}\right)$; and (3) feed the aligned window to the frozen classifier: $\hat{y}=A\left(x_{\mathrm{aligned}}\right)$.

### 2.8. Baselines for Fair Comparison

The primary reference metric is classifier *A* evaluated without any preprocessing (denoted “A-only”), quantifying naive cross-day degradation under frozen weights.

To disentangle structured alignment gains from depth or naïve adaptation confounders while keeping classifier *A* frozen and holdout splits untouched, we add three diagnostics: (i) SRM (ours) leveraging Stage S1 (F,C) plus Stage $S_{2}$ projector *P* optimized with Equation (15); (ii) Naive (src), an F+P front-end trained strictly on labeled source-days to test whether stacking capacity alone suffices; (iii) Naive (s+t-unl), the same topology trained jointly on labeled sources plus unlabeled adaptation days with light identity regularization (weight 0.01) yet no adversarial or MMD terms, isolating the value of unstructured target exposure versus SRM objectives.

### 2.9. Evaluation Protocol and Metrics

We evaluate in a subject-wise manner. For each subject, we train *A* on $D_{\mathrm{src}}$, learn SRM on $D_{\mathrm{src}}$ (labeled) and $D_{\mathrm{adapt}}$ (unlabeled), and report classification accuracy (%) on the holdout test days $D_{\mathrm{test}}$.

To assess robustness to stochastic training effects, we repeat the full pipeline with three random seeds (42, 4399, 114514) for each subject, resulting in 12 experimental runs (4 subjects × 3 seeds). For each subject, we report mean accuracy and standard deviation across the three seeds.

We report classification accuracy (%) on $D_{\mathrm{test}}$, averaged across seeds for each subject and across all subjects for overall performance. We additionally compute per-class accuracy and confusion matrices to diagnose failure modes.

Unless stated otherwise, we present exploratory descriptive statistics tailored to scarce subjects: repeated runs differ only in initialization seeds nested within subjects; hence, standard deviations illuminate optimization variance rather than population variance. Inferential superiority tests targeting clinical populations are deliberately avoided; pairwise win counts are summarized when helpful.

## 3. Results

We report holdout-day classification accuracy (%) under cross-day/session shift for four subjects, with three random seeds per subject (12 experimental runs total). Comparisons juxtapose SRM versus the primary baseline A-only alongside Naive (src) and Naive (s+t-unl) structural controls (Section 2.8). Means and standard deviations are computed over seeds for each subject—the spread primarily reflects stochastic training—not prespecified hypotheses at population scales. Methods keep classifier *A* frozen except where noted in later contrasts; Naive (src) and A-only use only labeled sources, whereas SRM and Naive (s+t-unl) consume unlabeled adaptation days.

### 3.1. Holdout-Day Accuracy Comparison

Table 1 provides per-subject holdout-day accuracy (mean ± standard deviation across three random seeds) for all methods. The results show pronounced subject-wise variability in cross-day performance degradation.

**Figure 5:**
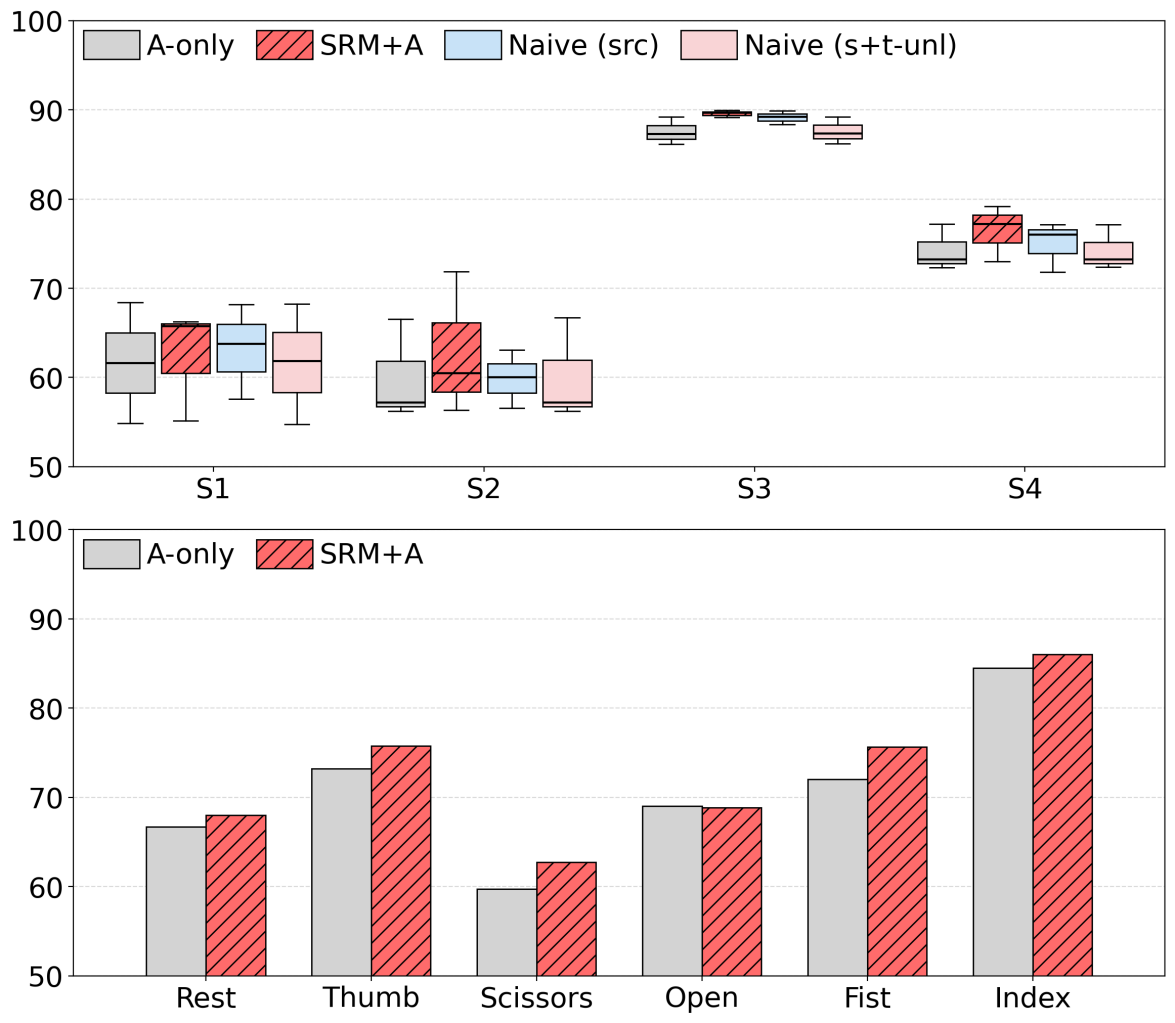
Holdout-day accuracy (%) on $D_{\mathrm{test}}$. (**Top**): per-subject box plots summarize only three random seeds (descriptive dispersion); we do *not* report uncorrected significance markers because *n* = 3 seeds per participant precludes dependable inferential conclusions (see statistical reporting in Section 2.9). (**Bottom**): mean per-gesture accuracy (12 runs) shows class-level gains for SRM+A versus A-only (e.g., Thumb, Fist, Scissors) while some confusions persist.

Figure 5 summarizes holdout-day performance in two views. The upper panel plots, for each subject, the distribution of holdout-day accuracy across the three random seeds as box plots, enabling comparison of all four methods (A-only, SRM+A, Naive (src), and Naive (s+t-unl)) while exposing seed-to-seed dispersion. The lower panel reports mean per-gesture accuracy on the holdout target domain, aggregated across all subjects and seeds (12 runs), contrasting A-only with SRM+A to show where alignment helps at the class level. Together with Table 1, these results show strong subject dependence: under A-only, the per-subject medians on holdout days span roughly 60% (Subject 2) to about 88% (Subject 3). SRM+A attains the highest median holdout accuracy for all four subjects in the upper panel. Relative to A-only, the largest mean improvements in Table 1 occur for Subject 2 (+2.9 percentage points) and Subject 4 (+2.2 percentage points). In the lower panel, SRM+A exceeds A-only on five of six gestures, with the largest margins on Thumb and Fist; Open remains nearly unchanged (both near ∼69%), whereas Index reaches the highest accuracy (∼86% with SRM+A) and Scissors remains the hardest class (∼60–63%).

### 3.2. Method Comparison and Analysis

The subject-wise results in Table 1 show a clear overall ranking. Averaged across subjects, SRM attains the highest holdout-day accuracy (72.8%), compared with 71.8% for Naive (src), 70.9% for Naive (s+t-unl), and 70.9% for A-only. Relative to A-only, the SRM gain ranges from about +0.7 percentage points for Subject 1 to +2.9 points for Subject 2, matching the subject-dependent severity of cross-day shift. Win rate denotes the fraction of runs where the proposed method outperforms each baseline. Across the 12 runs, the corresponding win rates over A-only are 58.3% (7 of 12 runs) for Naive (src), 41.7% (5 of 12) for Naive (s+t-unl), and 83.3% (10 of 12) for SRM.

The two naive baselines provide a useful contrast. Naive (src) improves over A-only for some subjects, but its mean performance remains below SRM. Naive (s+t-unl) adds exposure to unlabeled adaptation-day signals yet stays close to A-only at the aggregate level, indicating that target-day exposure alone is insufficient without an explicit alignment objective. Taken together, these comparisons support the benefit of combining unlabeled adaptation data with the structured two-stage alignment used by SRM.

### 3.3. Feature-Space and Class-Level Analyses

**Figure 6:**
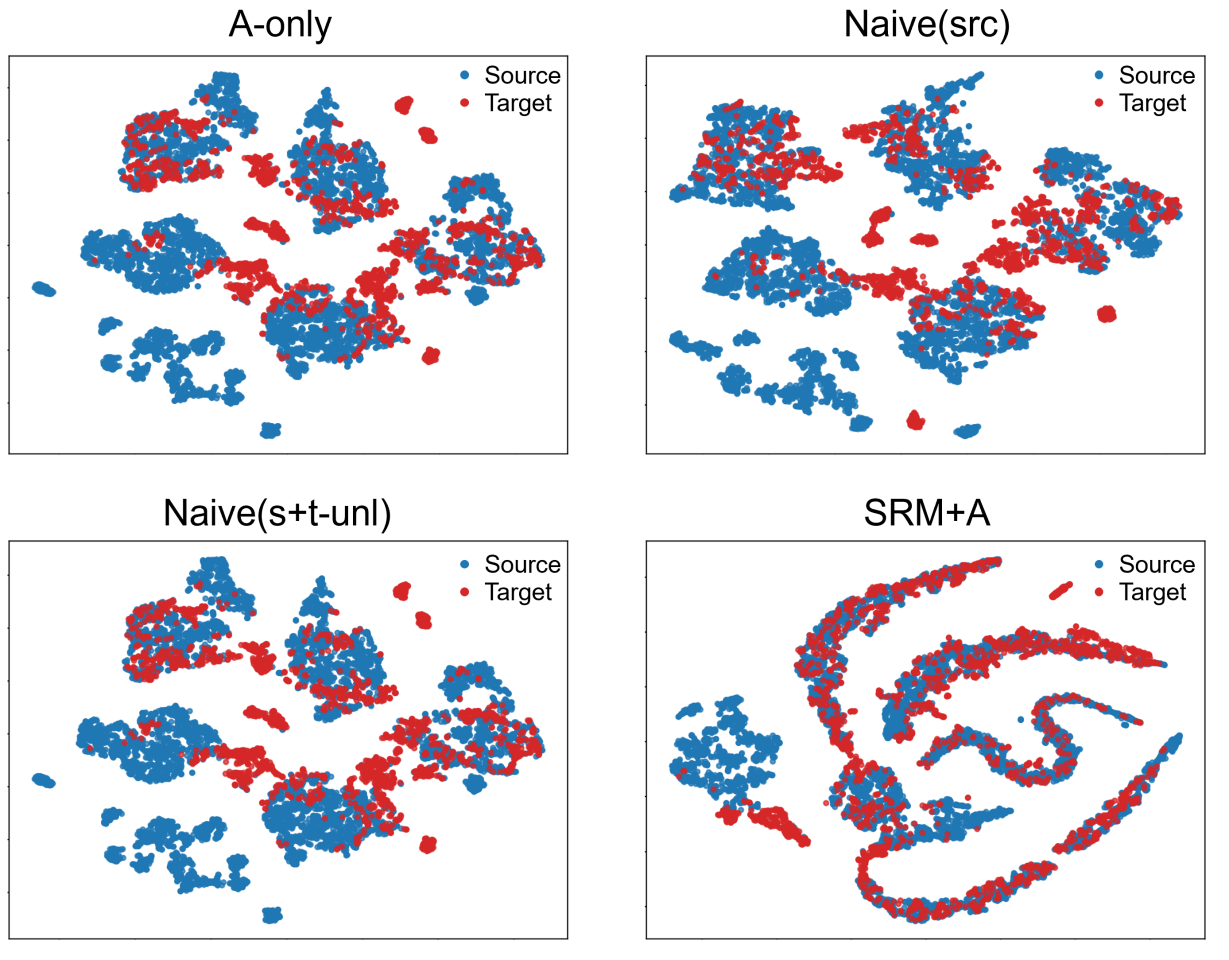
Illustrative domain-colored t-SNE [46] embeddings (Subject 2) computed on penultimate-layer features of frozen classifier *A*. Nonlinear embedding distorts global distances yet reveals qualitative domain overlap: sources (day-matched calibration) versus targets (evaluation days); SRM+A exhibits stronger inter-domain mixing versus baselines.

To provide visual evidence of the alignment effect, we visualize the penultimate-layer features of frozen classifier *A* using t-SNE [46], with samples colored by domain (source vs. target). Figure 6 presents this analysis for Subject 2 (low shift severity, +2.9% SRM gain) under four conditions: A-only, Naive (src), Naive (s+t-unl), and SRM+A.

Under A-only, source-domain samples and target-domain samples form largely disjoint clusters, indicating that cross-day shift appears as a systematic displacement in the penultimate feature space of classifier *A*. Naive (src), which is trained without any target exposure, reduces source–target separation only slightly and still leaves several target-dominated structures. Naive (s+t-unl) yields modest local overlap improvements, but the global separation remains readily apparent, consistent with its small aggregate gain over A-only in Table 1. After applying SRM, source and target samples become more mixed across gesture clusters. The reduced source–target separation in Figure 6 is consistent with projector *P* moving target-day signals closer to the source feature distribution expected by classifier *A*.

**Figure 7:**
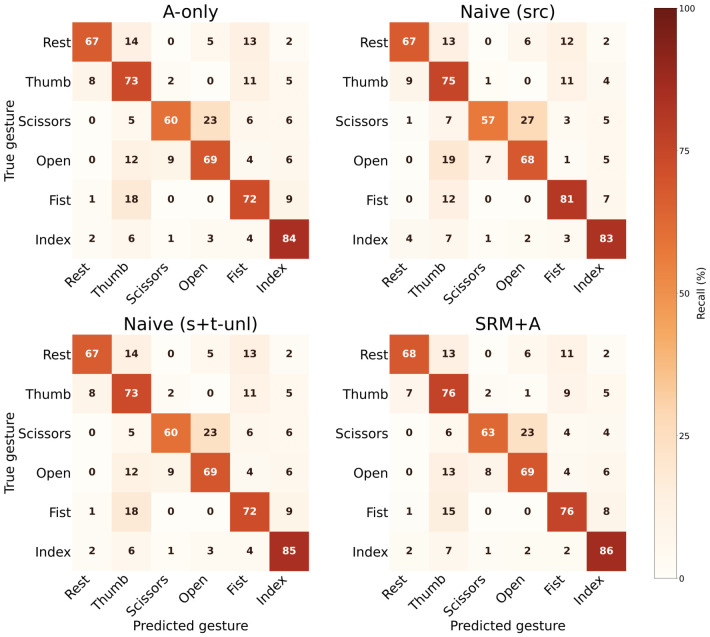
Row-normalized confusion matrices (%) aggregated over all four subjects and three seeds (12 runs) on holdout test days, for A-only, Naive (src), Naive (s+t-unl), and SRM+A. Diagonal entries are per-class recall; SRM+A improves recall on several gestures versus A-only, while Scissors–Open confusion remains a shared failure mode. Off-diagonal entries give misclassification fractions into each column class.

To examine whether this improved feature-space overlap translates into class-level recognition gains, Figure 7 shows row-normalized confusion matrices aggregated across all four subjects and three seeds (12 runs). Cross-day shift does not affect all gesture classes equally. Under A-only, per-class recall ranges from 60% (Scissors) to 84% (Index), with the most prominent error pattern being Scissors misclassified as Open (23%) and Fist misclassified as Thumb (18%), a persistent confusion attributable to the overlapping forearm extensor activation patterns of these gesture pairs.

Naive (src) and Naive (s+t-unl) differ markedly in their class-level behavior despite yielding similar aggregate accuracies. Naive (src) raises Fist recall from 72% to 81% but reduces Scissors recall from 60% to 57%, suggesting that source-only training shifts errors rather than eliminating them. Naive (s+t-unl) produces a diagonal pattern nearly identical to A-only across all six classes, confirming that unlabeled multi-domain exposure without a structured alignment objective provides no class-level benefit.

SRM shows the clearest aggregate gains across gesture classes: Thumb recall increases from 73% to 76%, Scissors from 60% to 63%, Fist from 72% to 76%, and Index from 84% to 86%. The Scissors–Open off-diagonal confusion remains near 23% for A-only, Naive (s+t-unl), and SRM+A, whereas Naive (src) increases it to about 27%, reflecting error reallocation under source-only training; nevertheless, the overall diagonal dominance is strengthened. Rest and Open recall remain stable across all four methods.

### 3.4. Comparison with Alternative Adaptation Strategies

We compare SRM against two common alternatives under the same six-class protocol, day splits, and three random seeds per subject (12 runs total): (i) end-to-end Conditional Domain Adversarial Network (CDAN), which jointly trains feature extractor *F* and classifier *A* with adversarial alignment, and (ii) supervised fine-tuning (Transfer Learning, TL), which loads a subject- and seed-matched copy of the main pipeline classifier, keeps the backbone fixed, and fine-tunes only the final fully connected layer on labeled holdout-day calibration data (first acquisition round) before evaluation on the same holdout test rounds.

For fair comparison, all methods use the same data splits: source days for initial training, adaptation days for domain adaptation (unlabeled for CDAN and SRM), and holdout days for calibration (Transfer Learning only) and final evaluation. CDAN [40,41,42] performs end-to-end training of feature extractor *F* and classifier *A* using labeled source data and unlabeled adaptation data via conditional adversarial alignment with gradient reversal, so both *F* and classifier *A* are updated during training. Transfer Learning [24,25,26] follows a “copy then recalibrate” protocol aligned with our implementation: for each subject and seed, we duplicate the classifier checkpoint saved by the main pipeline, load that copy without retraining the backbone from a fresh initialization, and fine-tune only the final fully connected layer on labeled samples from the first acquisition round on holdout days; evaluation then uses the same holdout test rounds as CDAN and SRM. This represents a conventional recalibration workflow when a brief labeled session on the deployment day is acceptable.

Table 2 summarizes per-subject holdout accuracy averaged over seeds (macro average over subjects) and the fraction of runs where each method exceeds the paired A-only baseline.

CDAN attains the highest macro-mean holdout accuracy (73.83%) but breaks the frozen-*A* assumption. Paired improvements versus A-only are highly dispersed (population standard deviation 12.4 percentage points across the twelve subject–seed runs), and CDAN beats A-only in 7/12 runs (58.3%), with subject-level means on S3 and especially S4 falling below the A-only reference despite strong gains on S1–S2. This is consistent with adversarial end-to-end alignment acting as a high-variance lever: it can lift performance when shift geometry matches the inductive bias, but it can also overshoot under unfavorable subjects or seeds.

With the same initial *A* as the main pipeline, Transfer Learning reaches 72.99% macro average, about 0.2 percentage points above SRM (72.83%), while requiring labeled calibration and updating classifier parameters (fc only). It exceeds A-only in 11/12 runs (91.7%) with the tightest spread of paired gains (standard deviation 1.4 points), i.e., slightly more run-to-run reliability against the frozen baseline than SRM in this grid. The near tie in macro mean versus SRM supports the narrative that much of the cross-day gap is not purely a decision-boundary issue, yet TL still pays a labeling and re-certification cost that SRM avoids.

SRM achieves 72.83% (+1.97 points over A-only) without target labels and without any update to *A*, wins in 10/12 runs (83.3%), and shows moderate paired-gain dispersion (2.3 points). Relative to CDAN, SRM trades about one point of macro mean for a 25 percentage-point higher win rate over A-only (83.3% vs. 58.3%) while keeping *A* immutable. Relative to TL, it sacrifices the small mean advantage and one fewer “win” over A-only in exchange for a strictly frozen classifier and label-free adaptation, which is the operating point targeted by this work.

Overall, these comparisons support a deployment-centric view: CDAN may maximize mean accuracy when full retraining is acceptable, Transfer Learning is attractive when a short labeled recalibration session is feasible, and SRM targets the constrained setting where neither option is available.

### 3.5. Ablation Study: Contribution of Individual Loss Components

The SRM objective (Equation (15)) combines three complementary terms: classifier-guidance loss $L_{A-\mathrm{CE}}$, identity preservation loss $L_{\mathrm{id}}$, and distribution-matching loss $L_{\mathrm{MMD}}$. To quantify the individual contribution of each component, we conduct an ablation study by independently training three degraded variants that each disable one loss group (NoMMD with $\lambda_{\mathrm{mmd}}=0$, NoID with $\lambda_{\mathrm{id}}=0$, and NoCE with $\lambda_{\mathrm{ce}}=0$) while keeping all other hyperparameters unchanged relative to the full objective.

We evaluate all four subjects (indexed as in Table 1) under the same six-class protocol, frozen classifier *A*, and holdout-day splits, using the same three random seeds (42, 4399, 114514) per subject. Table 3 reports holdout-day accuracy (%) for each subject as mean ± standard deviation over the three seeds (same convention as Table 1). Column A-only restates the frozen baseline under the identical evaluation; column All denotes the full SRM objective (the SRM column in Table 1); NoMMD, NoID, and NoCE each remove one loss term.

Table 3 supports three main observations. First, removing classifier guidance (NoCE) is the most damaging ablation in the aggregate mean row (59.8% vs. 72.8% for All): the per-subject means for NoCE fall below All for all four subjects (largest gaps for Subjects 2–4), and the reported standard deviations for Subjects 3 and 4 are large, indicating high seed-to-seed instability once $L_{A-\mathrm{CE}}$ is removed. This pattern indicates that, without gradients from frozen classifier *A*, projector *P* can pursue task-agnostic distributional alignment that is not compatible with *A*’s decision boundaries; the same qualitative failure mode discussed for Naive (s+t-unl) in Table 1, but more direct once $L_{A-\mathrm{CE}}$ is removed.

Second, identity preservation (NoID) illustrates a mean–reliability trade-off. At the bottom of Table 3, the aggregate mean for NoID (74.1%) is higher than that of the full objective All (72.8%) by about 1.3 percentage points, driven largely by large mean gains for Subjects 2 and 4. Yet this aggregate lift is not uniformly reliable: for Subjects 1 and 3, the mean NoID accuracy lies below the corresponding mean A-only accuracy (58.2% vs. 61.7% and 85.2% vs. 87.5%), i.e., removing $L_{\mathrm{id}}$ can push holdout performance under the frozen baseline on some individuals even though the cross-subject average rises. Counting paired subject–seed runs (four subjects × three seeds), NoID exceeds A-only in 7 of 12 runs (58.3%), whereas the full objective All exceeds A-only in 10 of 12 runs (83.3%). Thus, NoID attains a modest mean improvement relative to All at the cost of a 25 percentage-point reduction in win rate over A-only compared with the full SRM stack, a pattern more consistent with redistributing accuracy across subjects and seeds than with uniformly safer adaptation. We therefore interpret $L_{\mathrm{id}}$ not as an optional add-on that can be dropped for free mean gains, but as a stabilizer that helps keep per-run behavior aligned with the frozen classifier and the A-only reference, especially under heterogeneous cross-day shift.

Third, MMD (NoMMD) behaves as a mild, complementary regularizer rather than a uniformly dominant term. Its aggregate mean (72.5%) is close to All (72.8%), and per-subject means track All within about one point for Subjects 1–3, while slightly exceeding All for Subject 4 under the fixed weights. Head-to-head ordering between NoMMD and All is not monotone across the twelve runs, so we do not claim that MMD strictly increases holdout accuracy in every setting; instead, MMD provides distributional regularization that is most useful when paired with classifier guidance and identity scheduling.

Overall, the ablations support a layered story: $L_{A-\mathrm{CE}}$ is necessary for task-aligned, frozen-*A* adaptation; $L_{\mathrm{id}}$ trades off occasional large gains against baseline reliability; and $L_{\mathrm{MMD}}$ supplies auxiliary alignment whose benefit is context-dependent. These findings motivate subject- or seed-aware scheduling of $\lambda_{\mathrm{ce}}$, $\lambda_{\mathrm{id}}$, and $\lambda_{\mathrm{mmd}}$ as future work.

## 4. Discussion

### 4.1. Main Implications

Across the subject-wise experiments, the dominant failure mode is displacement of target-day samples away from the source feature distribution expected by *A*, consistent with cross-day changes in the acquired sEMG waveforms arising from electrode–skin and placement variability as discussed in Section 1. SRM partially corrects this displacement while leaving *A* unchanged, and the gain appears across overall accuracy, feature overlap, class-level confusion, and the ablation study.

The comparison with CDAN and Transfer Learning (Table 2) reinforces a deployment-centric reading. CDAN attains the highest macro-mean holdout accuracy in the twelve-run grid but retrains *F* and *A* and shows the largest dispersion in paired gains versus A-only, together with a lower win rate over the frozen baseline than SRM. Transfer Learning, loading the subject- and seed-matched main-pipeline classifier copy and fine-tuning only its final layer on labeled holdout-day calibration, achieves a marginally higher macro mean than SRM and a slightly higher win rate over A-only (11/12 vs. 10/12); SRM instead preserves *A* and performs label-free alignment upstream. Thus, SRM is most differentiated when neither classifier updates nor target labels are acceptable.

### 4.2. Deployment-Oriented Regulatory Positioning (Illustrative)

Disclaimer: interpretations below are methodological context only, not statutory advice, and jurisdiction-specific consultation remains essential.

Manufacturer-facing SaMD narratives often hinge on delineating immutable “locked” artefacts from configurable calibration modules. Throughout this manuscript, classifier *A* remains fixed after Stage A verification: its learned decision boundary is hashed and audited once. Conversely, projector *P* (with supporting frozen encoders trained off-line) behaves like exchangeable preprocessing whose updates may be staged through controlled tooling, analogously to repeatable calibration rigs used for EMG amplifiers. Unlabeled adaptation data collected after deployment resemble clinical fine-tuning of such front ends while leaving the audited classifier untouched. Any future practice that retrains *P* in the clinic would still belong to predetermined change-management procedures (risk analysis, versioning, usability validation) analogous to swapping approved filters or amplifiers; conversely, continuous cloud tuning without deterministic validation paths would exceed the reproducible safeguards studied here.

We therefore scope our contribution narrowly: quantify whether a deterministic SRM-training recipe can stabilize accuracy under annotated cross-day splits. Extending identical guarantees to networked updates is flagged as orthogonal engineering.

### 4.3. Generality and Potential Extensions

Although the experiments focus on multi-day sEMG gesture recognition, the framework may also be relevant to other tasks in which a validated classifier must remain fixed while inputs undergo distribution shift.

The core components of our framework (classifier guidance via cross-entropy loss, identity preservation via MSE regularization with warmup decay, and distribution matching via MMD or adversarial alignment) do not rely on sEMG-specific assumptions. The method requires only: (i) a differentiable frozen classifier *A* that provides gradient signals for aligning inputs, (ii) labeled source data and unlabeled target data, and (iii) an input space where residual corrections Δx can be meaningfully defined. Similar conditions may arise in other time-series classification tasks, including electroencephalography (EEG), electrocardiography (ECG), industrial sensor monitoring, and activity recognition from wearable accelerometers.

In principle, the framework could extend to image classification under domain shift (e.g., medical imaging with scanner variations, object recognition under lighting changes), provided the input-space residual $x_{\mathrm{aligned}}=x+P\left(x,z,\hat{p}\right)$ produces semantically meaningful transformations. However, image-level residuals may introduce artifacts or unrealistic perturbations, suggesting that feature-space alignment (inserting $S_{\mathrm{align}}$ between intermediate layers and the classifier) might be more suitable for high-dimensional visual data. For image tasks, inserting the alignment module in feature space is likely more appropriate than applying residual corrections directly in pixel space.

Our current protocol uses adaptation days to align with holdout test days. In practice, systems may encounter multiple target domains sequentially (e.g., weeks or months of use) or simultaneously (e.g., multiple users or devices). Two natural extensions are multi-day aggregation and online updates of $S_{\mathrm{align}}$ with explicit control of forgetting.

### 4.4. Limitations and Future Directions

Despite the demonstrated improvements, several limitations warrant discussion.

Our experiments are conducted on a proprietary sEMG dataset (MindRove ARB.210901, 8 channels, 6 gestures, ≥5 sessions per subject). The use of a custom dataset rather than a publicly available benchmark was motivated by two constraints inherent to the cross-day adaptation setting. First, the target deployment scenario for this framework is real-time myoelectric control using the same hardware pipeline as training; most established public benchmarks (e.g., NinaPro [35,36]) were acquired with different devices and electrode configurations, making direct transfer to our target hardware difficult and potentially confounding the evaluation of cross-day robustness. Second, and more fundamentally, the proposed framework requires that recording sessions be organized by acquisition date so that the day-split protocol can be constructed. To our knowledge, few publicly available sEMG corpora provide sufficiently dense multi-day recordings with explicit session timestamps per subject to support this protocol; those that do (e.g., some NinaPro subsets) typically contain only two or three sessions, which is insufficient for our five-day experimental design. Validation on public multi-session datasets with explicit day labels would show whether the observed gains generalize beyond the present hardware and cohort.

Our cohort (n=4) is small for drawing strong population-level conclusions about myoelectric decoding across users. All participants share young adult male physiology, excluding typical variation in adiposity and skin impedance seen across women, older adults, and many clinical populations experiencing muscle loss. The primary evidence target in this work is within-subject cross-day robustness under a frozen-classifier deployment constraint, where each subject contributes a multi-day split and repeated training seeds. Serving as a proof-of-mechanism, the four subjects are therefore intended as independent replications to mitigate concerns that improvements are idiosyncratic to a single user, rather than to estimate the prevalence of benefit in a broad population or to train a single universal model. Larger and more diverse cohorts (and, when feasible, multicenter acquisition) are needed to quantify inter-subject variability, failure modes, and subgroup effects with adequate statistical power.

The benefit of unsupervised adaptation depends critically on how representative the adaptation days are of the holdout days in terms of sensing conditions (e.g., band placement, skin impedance, perspiration, and residual motion relative to the source sessions). In cases where the adaptation days and the holdout days exhibit different types or magnitudes of domain shift relative to the source, adaptation may not generalize well. Selecting adaptation days by estimated domain proximity may reduce negative transfer when adaptation and holdout days differ substantially.

For Subjects 1 and 4, SRM performs comparably to the A-only baseline in certain seeds, indicating potential negative transfer under specific initialization conditions. Developing mechanisms to detect when alignment is beneficial (e.g., based on estimated domain gap or classifier confidence) and adaptively scale alignment strength could prevent degradation in such cases. A concrete next step is to gate or scale alignment using estimated domain gap or classifier confidence, so that SRM is not applied strongly when adaptation is likely to be harmful.

The ablation study in Section 3.5 covers all four subjects and three seeds per subject (Table 3). While this broadens empirical support, the relative importance of $L_{A-\mathrm{CE}}$, $L_{\mathrm{id}}$, and $L_{\mathrm{MMD}}$ varies markedly across individuals and initializations; the fixed loss-weight configuration ($\lambda_{\mathrm{ce}}=2.0$, $\lambda_{\mathrm{id}}=0.1$ with three-epoch warmup decay, $\lambda_{\mathrm{mmd}}=0.5$) is unlikely to be optimal everywhere. Subject-adaptive or validation-driven scheduling of $\lambda_{\mathrm{ce}}$, $\lambda_{\mathrm{id}}$, and $\lambda_{\mathrm{mmd}}$, as well as meta-learned selection, remain open directions for reducing run-to-run and subject-to-subject variability.

We evaluated the approach with three random seeds per subject, showing improvements over A-only in most runs (83.3% win rate across 12 runs, i.e., the fraction of runs where the proposed method outperforms the baseline). However, the standard deviation of performance differences varies across subjects and methods, indicating that adaptation benefit can be sensitive to initialization and stochastic training dynamics. Because the gain varies with initialization, a direct follow-up is to test whether ensembling or more stable initialization can reduce run-to-run variance.

## 5. Conclusions

In this work, we studied within-subject sEMG gesture recognition under realistic inter-day/session shifts and targeted a practical constraint: improving performance on a new day without retraining a pre-trained classifier and without requiring target labels. To this end, we introduced SRM that can be attached before a frozen classifier. The proposed training pipeline combines conditional adversarial learning to obtain a day-robust latent space with a residual signal-space projector that aligns raw inputs while being constrained by classifier guidance and identity preservation.

Across four subjects and three random seeds per subject, SRM improved holdout-day accuracy over the frozen baseline in 10 of 12 runs (83.3%), with a mean subject-level increment of 1.98±0.91 percentage points summarized across subjects (each subject averages three paired seed comparisons). Taken together, these results describe a reproducible exploratory trend favorable to SRM rather than generalized statistical confirmation against null hypotheses formulated at the population scale. Applying signal-space corrections upstream of immutable *A*, nonetheless cleanly separates transferable adaptation from rewiring classifier parameters.

Compared with surrogate strategies in Table 2, CDAN and TL can reclaim additional accuracy when policymakers permit parameter updates or targeted-day supervision; our contribution targets stricter regimes that forbid those freedoms. Closing the gap empirically will require diversified cohort studies integrated with clinically meaningful validation milestones.

## Figures and Tables

**Table 1:** Holdout-day accuracy (%) with classifier *A* frozen: SRM yields the highest subject-mean accuracy (72.8%) and wins over A-only in 10/12 subject–seed runs (83.3%) under the same splits (mean ± std over 3 seeds). Bold entries in the SRM column mark the highest accuracy per subject row.

Subject	A-Only	Naive (src)	Naive (s+t-unl)	SRM
1	61.7 ± 5.5	63.2 ± 4.3	61.6 ± 5.5	**62.4 ± 5.1**
2	60.0 ± 4.6	59.9 ± 2.7	60.0 ± 4.7	**62.9 ± 6.6**
3	87.5 ± 1.3	89.2 ± 0.6	87.6 ± 1.2	**89.6 ± 0.3**
4	74.2 ± 2.1	75.0 ± 2.3	74.2 ± 2.1	**76.5 ± 2.6**
Mean	70.9	71.8	70.9	**72.8**

**Table 2:** Comparison of cross-day adaptation methods under different deployment constraints. Bold entries in the SRM row highlight the proposed method under the frozen-classifier setting. The downward arrows (↓) indicate accuracy below the uncalibrated A-only baseline (negative transfer). Win rate: subject–seed runs where method > A-only (*n* = 12). The column Deployment Category highlights operational burden: “Strictly frozen” keeps classifier *A* immutable; “Unfrozen/higher labeling burden” groups methods that update parameters (CDAN) or consume labeled calibration on target days (TL), implying additional labeling or regulatory/documentation effort—not elevated physiological risk.

Deployment Category	Method	Update Params?	Target Labels?	S1	S2	S3	S4	Macro avg.	Win vs. A-Only
**Strictly Frozen** (Compliant/Safe)	A-only (Baseline)	No	No	61.65	59.98	87.54	74.24	70.86	-
	**SRM (Ours)**	**No**	**No**	**62.39**	**62.88**	**89.58**	**76.46**	**72.83**	**83.3% (10/12)**
**Unfrozen /** **higher labeling burden**	CDAN	Yes	No	74.81	75.15	83.95 **(↓)**	61.41 **(↓)**	73.83	58.3% (7/12)
	TL (Fine-tuning)	Yes	Yes	64.52	61.67	88.11	77.66	72.99	91.7% (11/12)

**Table 3:** Holdout-day accuracy (%) with frozen *A* (mean ± std over three seeds per subject). Bold column headers name ablation variants; bold numeric entries mark the best holdout accuracy per subject row. Columns denote: A-only, frozen backbone without preprocessing; All, full Equation (15) objective; NoMMD, disables $L_{\mathrm{MMD}}$ ($\lambda_{\mathrm{mmd}}=0$); NoID, disables $L_{\mathrm{id}}$; NoCE, disables $L_{A-\mathrm{CE}}$. Removing CE collapses aggregate mean accuracy (59.8% vs. 72.8% for All) with large seed dispersion for some subjects. Removing identity regularization lifts the pooled mean (74.1%) yet reduces reliability (wins over A-only in 7/12 versus 10/12 runs for All). Disabling MMD tracks All closely (72.5%).

Subject	A-Only	All	NoMMD	NoID	NoCE
1	61.7 ± 5.5	**62.4 ± 5.1**	61.6 ± 5.4	58.2 ± 1.9	61.7 ± 5.6
2	60.0 ± 4.6	**62.9 ± 6.6**	61.4 ± 6.2	72.7 ± 0.9	50.4 ± 8.0
3	87.5 ± 1.3	**89.6 ± 0.3**	89.1 ± 1.2	85.2 ± 1.4	68.2 ± 28.4
4	74.2 ± 2.1	**76.5 ± 2.6**	77.9 ± 1.2	80.2 ± 1.1	58.8 ± 22.7
Mean	70.9	**72.8**	72.5	74.1	59.8

## Data Availability

The software implementation used to produce the reported results is publicly available at https://github.com/Dianli97/ModuleB (accessed on 7 May 2026). The sEMG dataset is not publicly posted in accordance with informed consent and institutional policies. Access for reproducibility or meta-analysis may be requested from the corresponding author and will require ethics and institutional approval.

## Abbreviations

The following abbreviations are used in this manuscript:

CDAN	Conditional Domain Adversarial Network
ECG	Electrocardiography
EEG	Electroencephalography
FDA	U.S. Food and Drug Administration
GRL	Gradient reversal layer
MDR	European Union Medical Device Regulation
MMD	Maximum mean discrepancy
MSE	Mean squared error
SaMD	Software as a Medical Device
sEMG	Surface electromyography
SRM	Source-Reprojection Module
TL	Transfer Learning
t-SNE	t-distributed stochastic neighbor embedding
